# Traditional Conceptions of the Legal Person and Nonhuman Animals

**DOI:** 10.3390/ani12192590

**Published:** 2022-09-28

**Authors:** Macarena Montes Franceschini

**Affiliations:** Law Department, Universitat Pompeu Fabra, 08005 Barcelona, Spain; macarena.montes@upf.edu

**Keywords:** legal personhood, nonhuman animals, Kelsen, role, status, capacity, rights, duties, subject of rights

## Abstract

**Simple Summary:**

Every student comes across traditional conceptions of the legal person during law school. Professors usually explain that humans and corporations are legal persons, and that legal personhood is the most important category within the legal system as legal persons can enter into different legal relations, hold rights, and bear duties. Nonhuman animals are usually not mentioned in courses of this sort. This article examines four traditional concepts of the legal person and argues that nonhuman animals can be considered persons according to each concept. It notes that the law does not consider the concept of the human the same as the concept of the legal person, and that animals may benefit from an ecumenical defense, considering that legal practitioners such as judges commonly use these four traditional concepts of legal personhood, sometimes in the same ruling.

**Abstract:**

Since Roman law, the category of the legal person has been the most relevant legal category, allowing humans and entities to act within the law and enter into legal relations. The legal system does not consider nonhuman animals as legal persons but as property or as sentient beings regulated by the rules of property. Throughout history, there have been different concepts of the legal person, and some are still relevant today. This article examines four traditional concepts of legal personhood, arguing that nonhuman animals can be considered persons according to each concept. The article reaches three main conclusions. First, the legal person is not the same as the human. Second, the debate between the equivalence and the subset views poses a dilemma between a revolution or the reform of animals’ legal status. Third, an ecumenical defense of animal legal personhood may benefit animals as it supports animal persons according to any of the traditional concepts of legal personhood.

## 1. Introduction

Great confusion has surrounded the concept of the person as it has not only been examined in law but also in theology, philosophy, psychology, sociology, and anthropology. In law, there is great confusion because there are different concepts of the person. A law student will learn that the legal person can be anything the law says is a legal person, a subject of rights, or an entity with the capacity to hold rights or duties. These concepts of legal personhood do not exclude each other. In fact, judges sometimes reference several in the same ruling. This paper distinguishes and examines four traditional concepts of legal personhood that are still relevant today and argues that none imply that only a human and never a nonhuman animal (animal) can be considered a person in each of the specified senses:

(i) Personification of a set of norms.
(ii) Status or role.
(iii) Legal capacity to hold rights and bear duties.
(iv) Subject of rights.

I propose an ecumenical defense of animal legal personhood, arguing that animals can be considered legal persons according to all four traditional concepts of the legal person. Legal personhood can also be understood as a cluster concept, but I do not examine this concept in the article. An ecumenical defense of animal legal personhood may benefit animals for at least four reasons. First, legal practitioners such as judges commonly use these four traditional concepts of legal personhood, sometimes in the same ruling. Second, animal legal personhood has a greater chance of success by demonstrating that animals can be considered legal persons according to the four traditional definitions instead of choosing only one concept. Third, having several definitions of legal personhood may benefit animals because one concept may be more suitable in some cases, depending on the animal’s particular circumstances or characteristics. Fourth, demonstrating that animals can be considered legal persons according to any of these traditional concepts indicates that the case for animal legal personhood is strong, revealing to legal practitioners and the public that animal legal personhood is a serious claim. 

This article is structured as follows. Section 2 examines Kelsen’s concept of the legal person, understood as a personification of a set of norms. Section 3 examines the Roman law concept of the legal person as the status or role one plays in society. Section 4 examines the concept of the legal person as the capacity to hold rights and bear duties. Section 5 examines the concept of the legal person as the subject of rights, revealing a dilemma between the equivalence and the subset views. Finally, this article ends with a conclusion. 

## 2. Personification of a Set of Norms

Some think that individuals have certain rights independently of what other humans have done (natural rights), and some think that to say that an individual has rights only means that somebody has granted him or her those rights (positive rights). In other words, that all rights are equally artificial conventions. This position leads to the conception of legal personhood as a useful fiction or mere convention that needs not be related to any physical or metaphysical reality. 

Philosopher and legal scholar Hans Kelsen is the main exponent of this position. He proposed the *Pure Theory of Law* because he believed politics and morality were contaminating traditional legal philosophy, reducing the law to a social science [1] (p. 53). Kelsen claimed that the concept of the legal subject or the person is “simply an artificial aid to thought, a heuristic concept created by legal cognition—under the pressure of a personifying, anthropomorphic legal language—in order to illustrate the data to be dealt with.” [1] (p. 46). Hence, person is “simply a personifying expression for the unity of a bundle of legal obligations and legal rights, that is, the unity of a complex of norms.” [1] (p. 47). 

In other words, the legal person is simply a personification of a set of legal rules [2] (p. 26) or the meeting place for a set of rules [3] (p. 314). This concept of legal personhood has influenced contemporary authors, such as legal scholar Rafael Verdera, who defines the legal person as the meeting point and center for the assignment of rights and duties [4] (p. 202). Moreover, courts also use this concept of legal personhood. For example, in Cecilia’s case, judge María Alejandra Mauricio stated that: 

Most animals and, specifically, great apes are also made up of flesh and bones, are born, suffer, drink, play, sleep, have the capacity for abstraction, love, are gregarious, etc. Thus, the category of subject as the center for the imputation of norms (or “subject of rights”) would not only include the human being but also great apes—orangutans, gorillas, bonobos and chimpanzees. [5] 

It is undeniable that animals are the meeting point for a set of rules in our legal systems. Indeed, animal welfare, environmental, conservationist, and anticruelty regulations protect animals as individuals and species. Positivism supports animal legal personhood because anything can be a legal person as long as the law recognizes this status to an entity or being. Therefore, we can defend animal legal personhood using Kelsen’s theory.

## 3. Status or Role

Legal personhood understood as status originated in Roman law, where the legal person referred to the role played in society [6] (p. 751). People in Ancient Rome could be free or slaves; citizens or foreigners; *sui iuris* (free Roman women and men who were not subjected to the authority of the *paterfamilias*) or *alieni iuris* [3] (p. 307). Everyone had a status in Ancient Rome because it simply meant having a position within Roman society [6] (p. 752). Unfortunately, legal personhood as status was used to discriminate and exploit different groups within society.

Even though legal personhood as a status is mainly linked to Roman law, some scholars have continued using it during the 20th century [7] (p. 15) and 21st century [8] (p.53). This concept of legal personhood can be used to extend legal personhood to animals, rather than to exclude them. Indeed, people still have different statuses within society that are relevant to the law, such as being a citizen, resident, married, single, divorced, or heir, among others. Hence, legal personhood is like a mask or a hat that we employ to play different roles in the legal world as creditor or debtor, plaintiff or defendant, lessee or lessor, owner, or possessor [8] (p. 53). All these legal roles carry different rights and duties. Therefore, legal personhood as a status no longer indicates social classes to discriminate people but specific relevant roles people have within society that are relevant to the law. 

One cannot appeal to this understanding of legal personhood to attempt to exclude animals because animals can also play different roles. For example, they can be family members, they can be workers that eventually retire, they can be guides or assistants, they can be victims of illegal activities and natural disasters [9] (p. 1954–1955). Hence, I argue that animals can also be considered legal persons in the sense of status due to the different roles they play in society, which are relevant to the law. There are at least three arguments to defend that companion animals are legal persons in the sense of status: an anthropological argument, an empirical argument, and a legal argument. 

First, many people consider dogs, cats, and other domesticated animals as family members. During 2011, The Harris Poll of 2184 adults in the US determined that 91% considered their companion animal as a family member [10]. The same trend can be found in a national survey conducted among 1500 adults in the US in 2017, revealing that 94% considered their dogs as family members [11]. This survey also revealed the special bond that exists between people and their dogs. For instance, 56% say hello to their dog first when they come home and 54% would consider ending a romantic relationship if they believe their dog does not like their partner [11].

Second, psychological research has shown that people view animals as family members [12] (p. 550). In fact, people can be as attached to their dog as to their mothers, siblings, best friends, and significant others, and even closer to their dogs than to their fathers [13] (p. 261). Moreover, research on the bereavement process following the death of a companion animal has also confirmed that people and animals have such a close relationship that their death causes grief [14] (p. 267). These results confirm that companion animals play a significant role in society as family members. 

Third, the family is the basic social institution protected by law. For example, the Spanish Constitution ensures the family’s social, economic, and legal protection [15]. In Latin America, the 1980 Chilean Constitution [16] and the 1991 Colombian Constitution recognize the family as the fundamental institution of society [17]. Judges have also started to treat companion animals similar to children and, thus, examine shared custody and visitation in divorce cases [18] (p. 229). For example, on 27 May 2019, a Spanish court in Valladolid granted shared custody of dog Cachas, ordering that he should spend six months with each spouse, allowing visitations on weekends [19]. Therefore, the consideration of some animals as family members has pushed judges to apply family law to animals, acknowledging that their legal status as property is unsuitable for solving these types of cases. Furthermore, Law 17/2021 of 15 December 2021, which amended the Spanish Civil Code, now regulates companion animals’ shared custody and visitation in divorce and separation cases, ordering judges to consider the animal’s welfare when deciding these cases [20]. Not only are family law courts recognizing companion animals as family members. For instance, Argentine criminal judge Gustavo Daniel Castro recognized dog Tita, who was shot dead by a policeman, as a “nonhuman daughter” and recognized the plaintiff as Tita’s “father,” as well as recognizing Tita a subject of rights and nonhuman person [21]. Hence, courts worldwide are recognizing multispecies families, so undoubtedly, companion animals play a role in society relevant to the law as family members, specifically as nonhuman daughters or sons. 

Finally, legal personhood understood as status should not be necessarily limited to companion animals. Philosophers Sue Donaldson and Will Kymlicka propose a political theory of animal rights, which is based on the different relationships between humans and animals that generate distinctive rights and responsibilities [22] (p. 9). According to this theory, domesticated animals should be considered as full citizens because we have bred them to be interdependent with humans. Animals in the wild should be seen as separate sovereign communities and liminal opportunistic animals should be treated like migrants or denizens [22] (p. 14). Their specific rights and the duties humans have towards animals will depend on the status they have within society.

## 4. Legal Capacity to Hold Rights and Bear Duties

Most scholars claim that the legal person is a being that has the ability to hold rights and bear duties, thus considering legal personhood and the capacity to enjoy rights as synonyms [2] (p. 24). This view is a textbook definition of legal personhood [23] (p. 81). This section first examines the problems of considering legal personhood as the capacity to bear duties and then examines the problems of considering legal personhood as the capacity to hold rights in relation to animals. 

### 4.1. Problems with the Legal Capacity to Bear Duties 

Some scholars reject legal personhood for animals, arguing that as rights and duties are correlative, animals cannot hold rights because they cannot bear duties [24] (p. 66). I argue that duties are not a necessary condition for legal personhood for the following five reasons. 

First, rights and duties are not correlative because the possession of a legal right by someone does not entail the bearing of a legal duty by that same individual, rather it entails the bearing of a legal duty by someone else [25] (p. 42). According to this argument, if the law recognizes great apes the right to life that means that humans would have to bear the duty of abstaining from killing great apes. 

Second, even if holding a legal right entailed bearing a legal duty, animals could still hold rights because bearing duties is “simply to be placed under it” [25] (p. 41). Understanding a duty or a right is not always a condition for holding a duty or right, which guardianship proves. Thus, the law could place someone under a duty who does not understand that duty. 

I claim that there is an important difference between *bearing* a duty and *fulfilling* a duty. Animals, children, and intellectually disabled people can *bear* duties because the law can place them under a duty. However, as they cannot *fulfill* their duties due to different circumstances, such as immaturity, cognitive abilities, or illness, a guardian must act on their behalf. This difference is coherent with the legal distinction between the capacity to enjoy rights, understood as the ability to hold rights or duties (or to be placed under them), and the capacity to exercise those rights or duties on one’s own. The latter requires a guardian to act on one’s behalf if we cannot act on our own. 

For instance, if a chimpanzee receives a generous donation and the law contemplates a tax on donations, the chimpanzee will *bear* a duty, but the guardian will *fulfill* the duty and pay the tax on the chimp’s behalf. If the chimpanzee has offspring that need medical attention, although the donation was given to the chimp and not her offspring, since chimps have duties towards their infant, part of the funds should be re-directed to them. Hence, a guardian can fulfill duties on behalf of an animal, like an attorney or representative often fulfills duties on our behalf. 

Third, it is true that animals are not always aware of their duties, but sometimes they are, and sometimes we are not. Chimpanzees not only recognize duties to family members or to nonrelated members of a group but recognize duties of reciprocity to individuals who may not even be members of their species. Primatologist Frans de Waal shares a thought-provoking situation where two young chimps did not comply with their group duties. The chimpanzee group was given dinner when all group members came inside from the outdoor enclosure, but dinner got delayed because two chimps stayed outside longer enjoying the sunset and evening breeze. The rest of the group became agitated having to wait unusually long for their dinners because of these young rebels, so the two tardy chimps were put in a separate enclosure for protection. The next day, the rest of the chimps screamed angrily at the two tardy chimps [26] (p. 124), who showed signs of having understood perfectly why they were told off, not only with their body language, but by subsequently being the first in line at dinner time [27]. 

It is interesting that de Waal, who has gained international fame describing the life of chimpanzees employed in biomedical research, used to deny chimps had rights on the grounds that they did not have duties [28] (p. 215). Chimpanzees may not have duties towards humans, but according to de Waal himself, they have many duties towards other chimpanzees [29]. It was perhaps the realization of this incongruence that has led de Waal to drop the duty-based objection to chimpanzee rights [30] (pp. 178–179). De Waal currently holds a position that is very similar to that of advocates of chimpanzee rights, except he resists the use of the term *right* and speaks instead of our duties towards them [30] (p. 179). 

Fourth, in the past, Western legal systems recognized that animals could bear duties. Indeed, during the Middle Ages, animals legally bore duties and were trialed for breaching these obligations, such as abstaining from killing humans, destroying crops, or participating in zoophilia [31] (p. 280). Usually, courts convicted animals to death, but they sometimes acquitted animals for having a good character or not participating in the criminal activity [31] (p. 281). I certainly do not support animal trials, but they are useful reminders that humans once considered that animals could legally bear duties, so the legal system adapted to this conviction. Hence, our legal systems could accept that many animals bear duties in their societies or groups, recognizing that animals can be legal persons because they can also bear duties in their own unique ways. 

Fifth, the law, moreover, considers beings that cannot fulfill their duties, such as children, disabled individuals, and comatose or terminal patients, as legal persons. So, the idea that having duties is a requirement of legal personhood is simply false. However, the textbook definition of legal personhood as involving duties still confuses some judges. For instance, when the Nonhuman Rights Project (NhRP) filed a writ of *habeas corpus* (*habeas*) on behalf of chimpanzee Tommy in 2013, the Third Judicial Department of New York denied the petition, arguing that “unlike human beings, chimpanzees cannot bear any legal duties, submit to societal responsibilities or be held legally accountable for their actions.” [32] (p. 6). The Court based the ruling on the Black’s Law Dictionary definition of the person as a being capable of bearing rights and duties [33]. 

The source of the Black’s Law Dictionary definition of a legal person is the book *Salmond on Jurisprudence*, which defines persons as “any being whom the law rewards as capable of rights *or* duties” (emphasis added) [34] (p. 299), proving the dictionary’s definition to be mistaken. The NhRP argued that, as noted earlier, chimpanzees actually bear responsibilities within their communities and within chimpanzee–human communities, ostracize individuals who violate social norms, have a cooperative social life, and perform death-related duties [35] (p. 5). 

In any case, the State of New York Supreme Court, Appellate Division, Third Judicial Department’s argument is not the dominant trend among courts. In other *habeas* cases around the world, judges have argued that animals do not need to bear or fulfill duties, as Judge Luis Armando Tolosa argued in Chucho’s case [36]. The duties argument was not considered relevant in orangutan Sandra’s [37] [38] [39], chimp Cecilia’s [5], or woolly monkey Estrellita’s cases either [40]. 

In sum, the law currently recognizes certain beings that cannot fulfill their duties as legal persons, so the definition of legal personhood clearly includes these cases. Using the conjunction *and* suggests that the being must be capable of holding rights and fulfilling duties to be considered a legal person. On the contrary, using *or* clarifies that a legal person can hold rights but may not be capable of *fulfilling* its duties. Consequently, Salmond’s definition of legal person should be preferred to avoid this confusion.

### 4.2. Problems with the Legal Capacity to Hold Rights

According to the contemporary theory of rights, animals currently hold certain legal rights even though the law categorizes them as things and not as legal persons. Indeed, animals currently hold certain basic rights derived from animal welfare and anticruelty regulations, although these are weak rights that do not provide animals with the strong protection associated with legal rights [41] (p. 544). Some scholars criticize the definition of legal personhood as the capacity to hold rights precisely because it clashes with the contemporary theory of rights [42] (p. 4). 

Attorney Steven Wise, president of the NhRP, defines legal personhood as “the capacity to possess at least one legal right” [43] (p. 1). The NhRP files writs of *habeas corpus* on behalf of certain animals requesting courts to recognize an animal as a legal person with the capacity to hold the right to bodily liberty. Legal scholar Visa Kurki criticizes the NhRP’s litigation strategy due to employing this concept of legal personhood and identifies it as the “orthodox view of legal personhood” [44] (pp. 47–48). 

Kurki mainly criticizes two aspects of the NhRP’s litigation strategy. First, he claims that the NhRP grounds its strategy on animals lacking legal rights, so winning a case would turn them into legal persons with certain rights, framing the debate as “momentous and historic,” and thus, deterring courts [44] (p. 48). Second, he argues that animal advocates, specifically the NhRP, should center the debate on animals holding certain rights instead of legal personhood [44] (p. 48). I present five arguments in favor of the NhRP’s position. 

First, attaining fundamental rights for animals through courts or congress *is* momentous and historic. Animals have been exploited for centuries and have only been afforded basic (and weak) legal protection. Even though animals currently hold some basic legal rights, these are minimal, difficult to enforce, and allow animal suffering and abuse. The NhRP grounds its strategy on pushing the barrier and challenging the status quo, defying judges, and the public opinion to change the legal status of animals through litigation and activism, media presence, and education on animals’ cognitive abilities and ethology. It may be true that this strategy can shock some judges, but so has every expansion of rights throughout history. 

In fact, Latin American case law indicates that lawsuits on animal rights and legal personhood have not dissuaded judges. Quite the contrary, some courts have dared to consider great apes, bears, dogs, and monkeys as legal persons or subjects of rights. Higher courts such as Supreme Courts or Constitutional Courts have started selecting these cases for revision, considering them novel and an opportunity to rule on animal rights. Even if these courts finally dismiss these cases, they are showing interest and slowly granting more protections to animals. Animal legal personhood is becoming a familiar concept among legal practitioners and the public thanks to these lawsuits. 

Second, the only *entirely* successful *habeas* case in the world used the orthodox concept of the legal person to recognize chimpanzee Cecilia as a legal person [5]. Cecilia’s case is the only *entirely* successful *habeas* case in the sense that the *habeas* was granted, and not reversed by a higher court. However, there are other successful *habeas* cases for the animal rights movement. For example, woolly monkey Estrellita’s case was successful as the Constitutional Court recognized animals as subjects of rights protected by the rights of nature but denied the *habeas* because Estrellita had died. Going back to Cecilia’s case, judge Mauricio identified the concept of the legal person with the concept of the subject of rights, arguing that great apes are subjects of rights with the capacity for rights [5] (p. 33). Thus, the orthodox view of legal personhood benefited chimpanzee Cecilia, who is currently living in a sanctuary in Brazil [45].

Third, given that the judicial quest for animal personhood and animal rights is to improve matters for animals, we must ask ourselves the following question. Is the NhRP’s employment of the orthodox view self-defeating? Although the NhRP has not won a case in the US yet, we cannot ascribe this circumstance to the use of the orthodox concept of legal personhood. In fact, in chimpanzee Kiko’s case, the Fourth Judicial Department denied the petition, arguing that the *habeas* must challenge the confinement itself and seek immediate release from custody, rather than changing the confinement conditions [46]. Likewise, in the case of elephants Beulah, Minnie, and Karen, judge Bentivegna simply dismissed the case, arguing that the NhRP lacked standing as it did not have a significant relationship with the elephants and considered the case frivolous [47]. 

In chimpanzee Tommy’s case, the New York State Supreme Court, Appellate Division, Third Judicial Department dismissed the case, arguing that chimpanzees cannot hold duties [48]. Hence, the court dismissed this case using the orthodox view of legal personhood [44] (p. 54). In Happy’s case, among several other arguments, the court also argued that animals must bear duties to hold rights [49]. 

I consider that even if the NhRP had used a different concept of legal personhood in Tommy’s case instead of the orthodox concept of legal personhood, the court could still use the orthodox view to dismiss the *habeas*. First, courts are not forced to use the same concept of legal personhood that the plaintiff uses in their brief, so a court could prefer to use a different concept, considering—as this paper shows—that there are different definitions. Second, even if the NhRP decided to use a different definition, the court could still prefer to use a textbook definition of legal personhood as these definitions are familiar to legal practitioners. Thus, perhaps choosing a textbook definition of legal personhood is strategically convenient for the NhRP because it avoids having to additionally convince courts that the textbook definitions of legal personhood are wrong due to the contemporary theory of rights and that the court should use a different definition to rule that animals are legal persons. Third, even if the court had not used the orthodox definition of legal personhood, it could still argue that bearing duties is a necessary condition of legal personhood, considering that many believe that humans as a species have autonomy to bear duties [50] (p. 45). Throughout history, different concepts have influenced the concept of the person and seeped into the law, such as dignity, responsibility, and agency. Therefore, it is not strange for judges to link personhood and rights with duties, regardless of the chosen definition of legal personhood. Hence, attorneys must prepare for different scenarios, so the NhRP should challenge the duties argument in its briefs, considering it is a common argument against animals’ rights. 

Four, judges may not know about the contemporary theory of rights or may not apply philosophical theories to legal practice, so they may consider that animal welfare legislation and anticruelty statutes do not grant animals legal rights but rather regulate animals as objects of protection. For example, the Supreme Court’s Criminal Chamber in Chucho’s case considered that animals are sentient beings in Colombia, so humans have duties to protect animals derived from animal welfare legislation and case law, but this does not imply than animals have the right to welfare [51]. Some scholars consider that animals do not currently hold rights because they are still regulated as property, there are hardly any legal actions to protect their interests, and humans have certain duties toward animals as the law regulates them as objects of protection [52] (p. 32).

Fifth, Kurki suggests that the NhRP should avoid debating that the animal is a legal person, and instead focus on whether the animal is entitled to a specific right [44] (p. 58). In other words, arguing that the animal is a legal person is surplus to requirement. Considering the NhRP’s litigation strategy, Kurki’s proposal means proving that the animal has a right to bodily liberty through the *habeas*, without claiming that the animal is a legal person. Certainly, it would be easier to avoid having to convince courts that at least some animals are legal persons and directly argue that these animals are entitled to bodily liberty through the *habeas*. However, civil law and common law legal systems usually establish straightforwardly that *persons* are entitled to the *habeas*, forcing attorneys to prove that the animal *is* in fact a person during trials. In common law legal systems, case law, statutes, and famous legal dictionaries refer to the person when regulating or defining the *habeas*. In Tommy’s case, the NhRP referenced article 70 § 7002 (a) of the Civil Practice Law and Rules (CPLR) of the Consolidated Laws of New York in its *habeas* to demonstrate standing [53]. This statute regulates the *habeas* petition, clearly stating that persons may file the *habeas*: 

A person illegally imprisoned or otherwise restrained in his liberty within the state, or one acting on his behalf or a party in a child abuse proceeding subsequent to an order of the family court, may petition without notice for a writ of habeas corpus to inquire into the cause of such detention and for deliverance. A judge authorized to issue writs of habeas corpus having evidence, in a judicial proceeding before him, that any person is so detained shall, on his own initiative, issue a writ of habeas corpus for the relief of that person. 

The New York State Supreme Court (Fulton County) found that the term person under CPLR article 70 did not include chimpanzees and denied the petition [54]. Hence, courts do, in fact, examine the *habeas’* procedural requirements. If the NhRP omits proving that the animal is a person, the court could dismiss the case on procedural grounds for not complying with the legal requirements to file a *habeas*. The court’s refusal to consider a chimp a person under article 70 does not imply that the NhRP’s strategy is wrong. It just means that that specific court does not consider that a chimp complies with one of the legal requirements to file a *habeas*. 

Civil law legal systems also regulate the *habeas* through laws that detail who can file the writ. In these legal systems, judges must determine whether these requirements are fulfilled when deciding to grant or dismiss the *habeas*. In fact, in many countries the *habeas* law explicitly states that only physical persons can file the writ, so juridical persons cannot file *habeas*. For example, article 1 of Spanish Organic Law 6/1984, which regulates the *habeas* procedure, states that any illegally detained *person* will obtain the immediate availability of a judge [55]. Additionally, article 21 of the 1980 Chilean Constitution states that the *habeas* may be filed on behalf of a *person* whose right to personal liberty and individual security is illegally deprived, disturbed, or threatened [16]. Likewise, article 4 of Colombian Law 1095/2006 states that the *habeas* petition must state the name of the *person*, the date, and place where the *person* is deprived of freedom, and the name of the people who deprived the *person* of freedom [56]. Article 5 states that the judge will interview the *person* that has filed the *habeas* and article 6 states that the judge will order the *person’s* freedom [56]. 

Therefore, in civil law jurisdictions it is also relevant to argue that the animal is a person as *habeas* regulations state that the writ applies to *persons* and judges must apply the law, examining if the petitioner fulfills the legal requirements. This argument may apply to other fundamental rights that are specifically granted to *persons* in Constitutions, international treaties, and statutes. 

Considering that the concept of the person appears in statutes and case law on the *habeas* in different legal systems, attorneys representing animals may be forced to prove at least three things in court:

(i) The animal is a person.
(ii) The animal is entitled to the right to bodily liberty.
(iii) The animal’s imprisonment is unlawful.

Even though the orthodox concept of legal personhood can cause theoretical dilemmas, especially regarding the contemporary theories of rights, it does not necessarily produce the same dilemmas in court, where attorneys must evaluate what strategy has more chances of success. The textbook definition of legal personhood as the capacity to hold rights or duties may enable judges to decide that if an animal is entitled to some legal rights, then the animal has legal capacity and thus legal personhood, as occurred in Cecilia’s case. 

## 5. Subject of Rights

The legal person as the subject of rights is another classic textbook definition [57] (p. 9). As the subject of rights has the capacity to hold rights, the concept of legal personhood as a subject of rights is often confused with the previous concept of legal personhood as the legal capacity to hold rights or duties [3] (p. 311). This section first examines who can be a subject of rights and the following section examines the problems of this definition of legal personhood. 

### 5.1. Who Can Be a Subject of Rights? 

Rights need a subject, who is identified as the legal person [2] (p. 24). Scholars have argued for decades about who can be a subject of rights. On the one hand, the will theory links the capacity to choose or express one’s will to bearing rights, so only those who can express their will can hold rights. Therefore, this theory excludes children, the dead, people in a vegetative state, and people who due to their age or an intellectual disability cannot express their will [58] (p. 17). 

On the other hand, whether a being has a will of its own is irrelevant when deciding whom the law considers a subject of rights. The community creates subjects of rights when it recognizes a being or entity as a unit with interests deserving social protection [59] (p. 26). Thus, the interest theory argues that rights protect interests, and therefore, children, the elderly, and people with intellectual disabilities can hold rights. It is obvious that the interest theory of rights, which is very widely held in philosophy, makes the case for animal rights much easier than the will theory, or even, as some argue, directly supports animal rights [3] (p. 312).

This, however, does not mean that if the will theory is true, animal rights cannot be defended. It is unclear what it means to be able to choose, or if being able to choose certain things is enough to hold rights according to the will theory [58] (p. 18). Perhaps it means that at least some animals are able to choose [58] (p. 18). The following three examples demonstrate animals’ ability to choose:

(i) Animal welfare preference tests.
(ii) Primatological research on capuchin monkeys, sooty mangabeys, and chimps.
(iii) Observation of orcas’ behavior in the wild.

First, animal welfare scientists normally discuss animals’ preferences, desires, and motivations when assessing an animal’s welfare through preference tests [60] (p. 31). During these tests, the animal has to choose between different options or environments, such as temperature, illumination, bedding, and flooring [61] (p. 159), as well as whom the animal prefers to approach and under what conditions [62] (p. 349). Furthermore, animal welfare scientists have also suggested that animals’ motivation is not limited to obtaining desirable outcomes and avoiding undesirable ones but they are also motivated to learn and manage the world around them [63] (p. 7).

Second, primatologists have also observed apes’ ability to choose, sometimes through unethical experiments. For instance, research has shown that capuchin monkeys refused to eat when the lever that allowed them to receive food gave another monkey an electric shock. Some monkeys did not eat for twelve days to prevent other monkeys from suffering [64] (p. 178). Chimpanzees are also capable of choosing. They develop complex social relations that imply choosing allies and defining the advantages and disadvantages of different options [64] (p. 71). Another study on sooty mangabeys and chimps shows the complex decision-making process in grooming. Both species had to choose a partner from a group of available individuals. These animals had to take the social environment into account before selecting a partner, such as avoiding grooming an individual that had strong social relationships with another bystander and considering the available partners’ rank in their decision [65] (p. 9). Other examples of animal choice provided by de Waal involving chimpanzees and dolphins convinced choice theorist Hillel Steiner that some animals should have rights [58] (p. 19). 

Chimpanzees show both culturally influenced preferences and idiosyncratic preferences that have no adaptive explanation. For example, even if building a nest at 3 m is enough to obtain security against predators, chimpanzees of different areas build at different heights, ranging from 3 to 45 m, and within each group some individuals make the extra effort involved in building at enormous altitudes for no apparent reasons but sheer taste [66] (p. 114), [30] (p. 219).

Third, the case of orcas is particularly striking because while all orcas are remarkably similar and can benefit from eating other mammals, some entire populations do not do so and tend to avoid those who do [67] (p. 705). Even some individual members of the populations which sometimes eat mammals can refrain from doing so. For example, on 10 January 2022, witnesses caught on video a pod of orcas freeing a trapped humpback whale off the Western Australian south coast near Bremer Bay. Even though Bremer Bay orcas often eat humpback whales, and the tangled humpback whale would have been an easy meal, the orcas decided to free the whale from the entangled rope [68].

Confronted with the choice between the will and interest theory of rights, I would, I think with most, find the latter most plausible. Some, however, have responded to the choice theory, attempting to combine the two. Legal scholar Alexander Nékám, for example, argues that every right needs an administrator, who can only be a human capable of expressing her will. The subject or beneficiary of the right, however, can be any being or entity, which the community considers a unit with significant interests that require legal protection [59] (p. 33). In other words, only administrators (e.g., guardians) must be able to express their wills, while subjects of rights merely need to possess interests that the community deems essential to protect. The legal system reflects this position because it requires representatives and guardians to be paradigmatic adults who can express their will but does not require the wards to express their wills. As wards are vulnerable due to immaturity, illness, or cognitive abilities, they require the protection of their interests through a guardian or representative. 

### 5.2. Problems with the Subject of Rights View 

There are two main criticisms to the conception of legal personhood as merely entailing a subject of rights. First, some reject identifying legal personhood with being the subject of rights because this concept overlooks the passive function of personhood related to bearing duties and responsibility, and makes personhood depend on the number of rights a being gains or loses [2] (p. 29). This objection seems question begging to me, as it is far from obvious that there is something wrong with being guided by the number of recognized rights an individual or group of individuals possess. 

Second, Kurki’s criticism that the orthodox view of legal personhood fails to explain the contemporary theory of rights also applies to the concept of the legal person as a subject of rights. Indeed, animals are currently subjects of rights because they hold certain rights but are not considered legal persons. Adopting a conception of legal personhood that takes rights possession as a sufficient condition for personhood may be beneficial to animals as it may enable judges to consider animals as legal persons, if they also grant them some rights, as happened in chimp Cecilia’s and orangutan Sandra’s cases. 

In Sandra’s case, judge Elena Liberatori (a premonitory name) actually made the inverse inference, claiming that Sandra was a person and therefore a right holder: “nonhuman person, thereby, a subject of rights […] Sandra’s classification as a ‘nonhuman person,’ and in consequence, a subject of rights […]” [37] (pp. 6–7). In Cecilia’s case, judge Mauricio argued that the law identifies the concept of the person with the concept of subject of rights and declared great apes in general, and Cecilia, in particular, as subjects of rights [5] (p. 31). Mass media made Cecilia and Sandra famous as the first nonhuman natural legal *persons* in history [69] [70]. 

Are the legal person and the subject of rights interchangeable concepts? If so (let us call this the equivalence view), does it matter if animals are considered one thing or the other? Those who hold the equivalence view think that whenever a judge recognizes that an animal has some rights, the judge is granting the animal legal personhood as occurred in Sandra and Cecilia’s cases. Others believe that only some subjects of rights (humans) are, additionally, persons [3] (p. 318], and some believe that animals are subjects of rights but not legal persons [71] (p. 67), [72] (p. 324). 

At this point, one may think. Oh well, what’s in a word? It does not really matter what label we use, so long as they receive the proper protections. But the label is not entirely inconsequential. Replacing the equivalence view with the view that having rights is necessary but not sufficient for legal personhood (let us call this the subset view) may not be beneficial to animals for three reasons. 

First, the subset view leads to creating an intermediate category for animals, where animals may hold some rights, most likely weak rights [41] (p. 544), derived from animal welfare and anticruelty provisions as this has been the common trend worldwide. If the only rights granted to animals are related to welfare, and anticruelty provisions, recognizing animals as subjects of rights will coexist with their regulation as property, as animals can be regulated as property while holding some basic rights [73]. In short, animals’ situation will not really change in this scenario. 

Second, including all humans born alive and separated from the mother into the same category (physical persons) implies excluding other beings from that category, stressing the sharp contrast between humans and other beings [74] (p. 23). This places humans in a higher legal category so human interests would continue generally trumping animal interests. This is the legal equivalent of a position in moral philosophy held by many self-declared antispeciesists, such as philosophers Peter Vallentyne [75] and Shelly Kagan [76], who accord animals a lower moral status than they accord humans, declaring all their interests as having lesser moral importance. Persons have had the highest legal status since antiquity, so putting all animals in a different legal category than humans supports human exceptionalism.

Third, even though sentient animals have far more in common with humans than with nonsentient beings [77] (p. 68), the category of subjects of rights could include animals, and other nonsentient beings that also hold legal rights, such as nature and its elements, such as rivers, mangroves, and forests, which have been recognized as subjects of rights in different countries from India, Bangladesh, and New Zealand to Colombia and Ecuador. This category could also include other nonsentient beings such as idols or ships [78] (p. 138), which are not legally protected due to their vulnerability like humans and animals but due to other reasons such as worship or business. 

**Figure 1:**
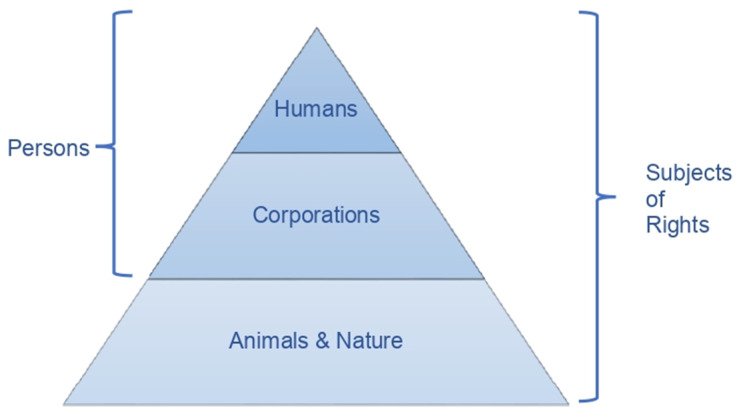
The Subset View.

**Figure 2:**
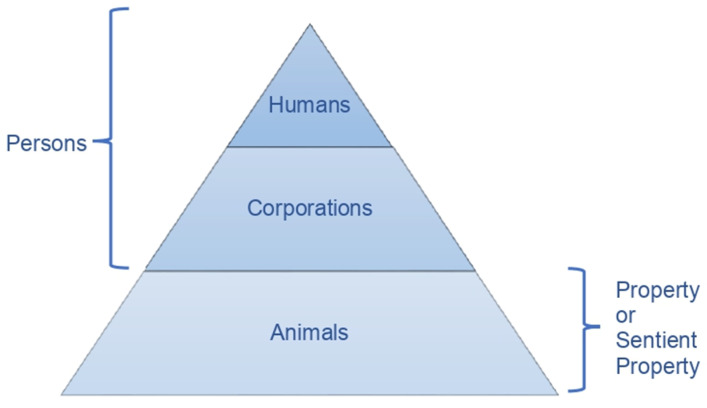
Animals’ Current Legal Regulation.

The subset view leads to the following organization of the legal universe (Figure 1):

An important variation on this model involves classifying corporations as subjects of rights but not persons, because only humans are legal persons [79] (p. 234). The subset view looks similar to how the law regulates animals today in most countries, as the next diagram shows (Figure 2): 

As explained above, the equivalence view makes animal legal personhood more achievable as holding rights is considered a sufficient condition for personhood, helping to break the personhood barrier, and expanding the movement and support for animal persons. However, the subset view is currently more acceptable for legal practitioners because it separates humans from other animals and maintains humans in the highest legal category, creating an intermediate category for animals. So, a dilemma emerges. Should we choose between a revolution or a reform of animals’ legal status? The subset view may be more acceptable for legal practitioners in the short term, but we should also strive to include at least some animals in the highest legal category with humans, so their interests can trump humans’ interests and they can enjoy the fundamental rights commonly granted to physical legal persons and the procedural guarantees that ensure those fundamental rights.

In sum, according to the will and interest theories, animals can be considered subjects of rights. However, the law protects vulnerable people’s interests, such as children and intellectually disabled people, rather than protecting only those who can express their will. The law even protects animals through anticruelty, welfare, and environmental regulations, supporting the argument that Western legal systems adopt the interest theory instead of the will theory [77] (p. 43). Traditionally, the legal person has been understood as the subject of rights, but in recent years a trend that separates these concepts has emerged. The subset view may be more acceptable to legal practitioners in the short term, but the equivalence view applied to animals strives for a revolution by breaking the legal personhood barrier that separates humans from other sentient animals.

## 6. Conclusions

The concept of the person has been examined in different fields such as psychology, theology, and philosophy, making it hard to define. Throughout history, different conceptions of the legal person have emerged. Some have become common among legal practitioners. I propose an ecumenical defense of animal legal personhood, arguing that animals can be considered legal persons according to four traditional concepts of legal personhood:

(i) Legal personhood as the personification of a set of norms.
(ii) Legal personhood as status or role.
(iii) Legal personhood as the legal capacity to hold rights and bear duties.
(iv) Legal personhood as the subject of rights.

These concepts vary depending on one’s theoretical framework, the role of the entity (or being), legal capacity, and holding rights. Throughout history, completely different entities, including idols, ships, corporations, charities, animals, rivers, mangroves, forests, and even nature in general, have been considered somewhere a legal person. It is thus unsurprising that the definition of what that means has significantly varied too.

If we follow Kelsen’s theory and embrace positivism, animals can be legal persons, as can anything the law recognizes as such. Roman law is responsible for developing the concept of the legal person as the different roles we play in society. Currently, animals play important roles within our societies, as family members or according to Donaldson and Kymlicka, as citizens, denizens, or migrants [22]. Animals can also possess the capacity to hold rights, and even though duties are not a necessary condition for legal personhood, many animals bear duties within their communities or can be put under a duty by the law. According to the interest and will theories, animals can also be considered subjects of rights.

Despite the variety of definitions of legal personhood and the confusions some of them originate, we can draw three conclusions. First, the law does not consider the terms human and legal person as the same. The concept of the human is a biological category that indicates who belongs to the *Homo sapiens* species, which can be determined through genetic testing. Second, the debate regarding the equivalence and the subset views of the subject of rights poses a dilemma between a revolution or the reform of animals’ legal status. Even though we may be able to advance animals’ legal protection in the short term by choosing the subset view as it does not challenge human exceptionalism, we should strive to include at least some animals in the physical legal person category with humans so their interests can trump humans’ interests. Third, an ecumenical defense of animal legal personhood may benefit animals as it supports animal persons according to any of the traditional concepts of legal personhood, considering that all these concepts are commonly used by legal practitioners, sometimes even in the same ruling.

## Data Availability

The data presented in this study are available on request from the corresponding author. The data are not publicly available due to privacy.
